# Mucinous epidermoid carcinoma of the lung with ALK mutation: Case report and literature review

**DOI:** 10.1097/MD.0000000000038301

**Published:** 2024-05-31

**Authors:** Lijiao Yang, Yanyan Ren, Xing Yin

**Affiliations:** a Department of Oncology, Handan First Hospital, Handan City, China.

**Keywords:** ALK, lung cancer, mucoepidermoid carcinoma, targeted therapy

## Abstract

**Rationale:**

Pulmonary mucoepidermoid carcinoma (PMEC) is a rare lung malignancy, especially in combination with ALK mutations, whose clinical presentation lacks specificity and for which there are no standardized treatment guidelines.

**Patient concerns:**

We report a case of a patient with PMEC-predominant primary lung cancer combined with an ALK mutation.

**Diagnoses:**

One patient was diagnosed with PMEC combined with ALK mutation.

**Interventions:**

After diagnosis by puncture pathology, the patient was treated with oral targeted drugs.

**Outcomes:**

The patient’s cough and fever were controlled, her diet improved significantly, and she gained 20 pounds in 6 months. During this period, the primary and metastatic foci in the lungs were significantly reduced on repeat chest CT.

**Conclusion:**

PMEC combined with ALK mutation is an extremely rare primary lung cancer, and the diagnosis is mainly based on pathology, histology and immunohistochemistry. The application of molecularly targeted drugs to patients with mutations can significantly improve the prognosis of patients with PMEC, which is expected to be a new breakthrough in the treatment of PMEC.

## 1. Introduction

Mucoepidermoid carcinoma (MEC) is an invasive malignant tumor commonly found in the salivary or lacrimal glands, whereas pulmonary mucoepidermoid carcinoma (PMEC) is rare. It is usually thought to originate from the ductal epithelial cells of the tracheal and bronchial submucosal glands, and its incidence accounts for 0.2% of primary lung tumors.^[1,2]^ PMEC has atypical clinical features, complex pathologic and histologic components, and difficulty in preoperative diagnosis, and its traditional treatment is single, mainly surgical treatment, and the effect of adjuvant radiotherapy is still unsatisfactory.^[3]^ Currently, the research progress of molecular targeted therapy is in full swing, and immunotherapy has opened a new chapter in human tumor treatment, but there is less experience in targeted therapy and immunotherapy for PMEC. Due to the low incidence of PMEC, there is less literature at home and abroad, and most of them are case reports, so there is a lack of summary of its clinical manifestations, pathologic features as well as histologic features, imaging features, clinical diagnosis and treatment, and prognosis. By reviewing the relevant literature on targeted therapy for PMEC, it is hypothesized that targeted therapy may be a new breakthrough in the treatment of PMEC. In this paper, we report a case of PMEC-based primary lung cancer combined with ALK mutation, and discuss the key issues of clinical diagnosis and treatment in the light of relevant literature review.

## 2. Case report

### 2.1. Chief complaints

A 57-year-old woman presented on October 4, 2022 with “coughing discomfort for half a month and a 2-day history of right lung occupancy.”

### 2.2. History of present illness

The patient was admitted to the hospital half a month ago without any cause cough, dry cough, accompanied by low-grade fever, the local hospital anti-infective treatment did not improve, chest CT examination suggests: solid nodules in the upper lobe of the right lung (22 × 15 mm), consider the tumor lesion, the mediastinum and the right hilar multiple enlarged lymph nodes (the large one is 3.4 cm in diameter), consider the metastasis. The patient was referred to our hospital for further assessment.

### 2.3. History of past illness

He past medical history was healthy.

### 2.4. Personal and family history

There was no family history of malignancy or any other family history.

### 2.5. Physical examination

Upon physical examination, Cardiopulmonary and abdominal examination showed no positive signs.

### 2.6. Laboratory examinations

Routine blood examination, coagulation function, urinalysis results, stool analysis, liver chemistry tests, urea, creatinine, uric acid and electrocardiogram results were all within normal limits.

### 2.7. Imaging examinations

**Figure 1.**
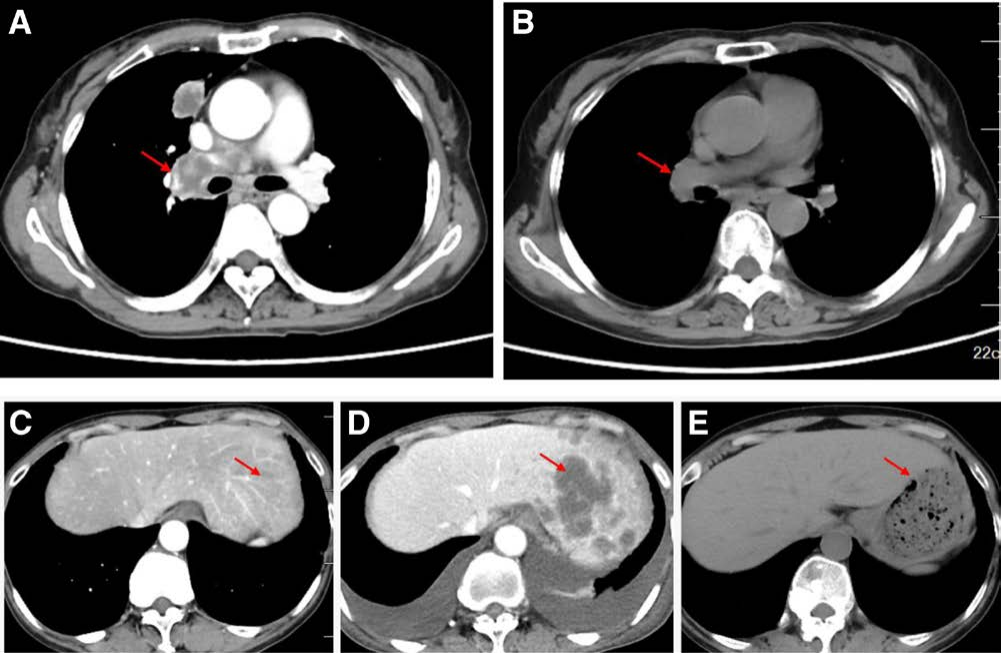
(A) Pretreatment lung lesions; (B) 2023-12-8 ALK orally for 6 months; (C) 2022-10-7 Intrahepatic condition at initial diagnosis; (D) 2023-4-3 Intrahepatic metastases after chemotherapy progression; (E): 2023-12-8 Intrahepatic lesions at 6 months of oral ALK.

Chest CT (enhancement): Solid nodule in the anterior segment of the upper lobe of the right lung, size about 22 × 15 mm, enhancement is ring-shaped enhancement, consider tumor lesion, malignant possibility; Pure ground-glass nodule in the lateral segment of the middle lobe of the right lung, size about 14 × 10 mm, adjacent pleura is stretched, tumor lesion is not excluded; Multiple enlarged lymph nodes in the mediastinum and the right hilum, the large one is 3.4 cm in diameter, enhancement is Ring-shaped enhancement, consider metastasis; Left adrenal soft tissue density shadow, consider metastasis; Multiple nodular abnormal enhancement shadows in the left lobe of the liver, consider metastasis (Fig. 1).

### 2.8. Pathologic findings

**Figure 2.**
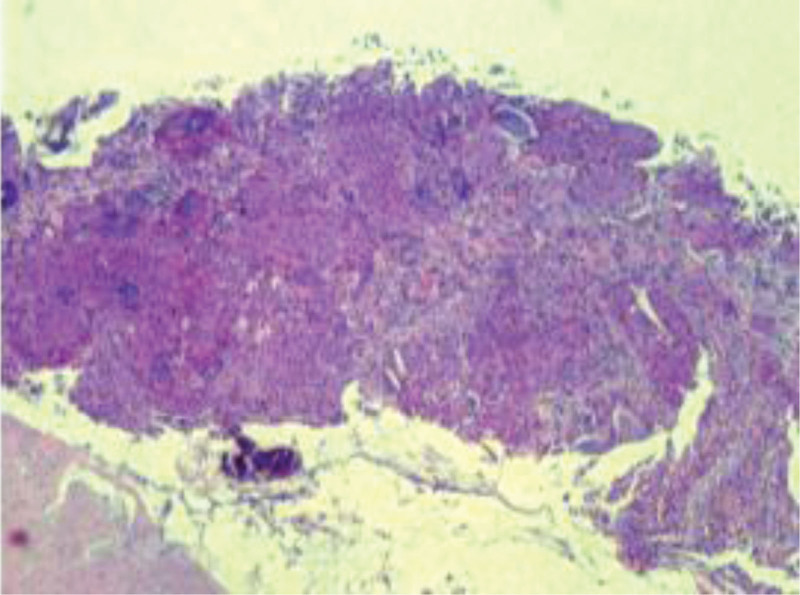
Hematoxylin-eosin staining of biopsy specimens indicated Mucoepidermoid carcinoma.

CT-guided aspiration biopsy of right lung mass was performed on 2022-10-14, Pathology: (right lung) poorly differentiated carcinoma, inclined to mucoepidermoid carcinoma. Immunohistochemistry: CK7 (partially +) CK20 (-) CDX-2 (-) Ki-67 (+ about 40%) P40 (partially +) TTF-1 (-) CK (+) PD-L1 (SP263) (TPS about 2%) (Fig. 2).

### 2.9. Final diagnosis

PMEC was diagnosed at stage IV (cT1cN2M1), according to the NCCN guidelines (2022).

### 2.10. Treatment

TP (albumin paclitaxel + nedaplatin) combined with bevacizumab antivascular targeting and teraplizumab immunotherapy was given, and chest and abdominal CT was reviewed after 4 cycles, suggesting: 17 × 14 mm nodule in the anterior segment of the upper lobe of the right lung, with little change; Pure ground-glass nodule in the lateral segment of the right middle lobe of the right lung, 14 × 10 mm; Multiple enlarged lymph nodes in the mediastinum and the right hilar region of the lungs, with a large one of 3.3 cm, which was larger enlarged than before; Multiple hypodensities in the liver, increased and enlarged than before; Multiple enlarged lymph nodes in the hepatogastric hiatus and retroperitoneum, which were considered to be metastatic (Fig. 1). The efficacy was assessed as progressive disease (PD), and the patient was in poor general condition, with marked emaciation, persistent hypothermia, and could not tolerate continuation of chemotherapy. Intrahepatic lesion aspiration biopsy was performed on 2023-4-7, genetic testing: EML4-ALK E6A20 5.7%, PDL-1 < 1%. Oral loratinib was initiated to date, and the patient’s symptoms such as cough and fever were controlled, her diet improved significantly, and she gained 20 pounds in 6 months. During this period, the primary and metastatic lesions in the lungs were significantly reduced in the follow-up chest CT, and the efficacy of the treatment was evaluated as partial response (PR).

### 2.11. Outcome and follow-up

The patient has been taking oral loratinib since then. At the time of writing (2023-12), efficacy was assessed as partial remission (PR, Fig. 1).

## 3. Discussion

PMEC is a rare lung malignancy, which can be classified into low-grade mucoepidermoid carcinoma and high-grade mucoepidermoid carcinoma according to the ratio of the composition of mucous cells, epidermoid cells and intermediate cells. Low-grade malignant tumors are characterized by cystic components, and high-grade malignant tumors are characterized by atypical cells, nuclear schizophrenia and necrotic manifestations and regional lymph node metastases.^[3]^ Mucinous epidermoid carcinoma of the lung was first reported by Smetana in 1952, and the data showed that PMEC was commonly seen in 30 to 40 years of age, with a predominance of male patients, but the association with smoking was not significant, and it occurred in children and young adults.^[4]^

In general, patients with tumors located in the hilar region of the lung will have symptoms of bronchial obstruction, such as cough, dyspnea, wheezing and hemoptysis, of which cough is the most common clinical symptom, and about 70% of patients with bronchial MEC present with cough, while patients with tumors located in the peripheral sites may have chest pain, cough or pneumonia.^[5,6]^ The clinical symptoms of mucoepidermoid carcinoma of the lung are not specific, and are mainly related to the location and size of the tumor, the invasion of the airways and the degree of airway obstruction.^[7]^ Mucoepidermoid carcinoma of the lung mainly appears as an isolated tumor mass on CT, which can occur in any part of the lung, and in the main and lobar bronchial foci, it can be seen as a rounded or lobulated mass, which can be accompanied by obstructive pneumonia or pulmonary atelectasis, the density of the mass is relatively homogeneous, and enhancement scans show mild enhancement, and some of them are combined with intra-tumor calcification.^[8]^ The lung CT image of our patient is more consistent with this pattern. PMEC has a low incidence rate and is not specific in clinical manifestations and imaging, so the diagnosis is mainly based on histopathology, and its histological components are complex, which can be diagnosed with the help of immunohistochemical results. It has been found that cancer cells of PMEC can express CK8/18, CK5/6, CKpan and NapsinA; P63 cancer nuclei were positive, TTF-1 and SMA were negative; Ki-67 positive cells are 2% to 40%, and mucinous cells are positive for AB/PAS staining, and these immunohistochemical findings can help us distinguish it from squamous cell carcinoma, adenosquamous cell carcinoma, and adenoid cystic carcinoma.^[9]^

Although there is no uniform standard for the treatment of mucoepidermoid carcinoma, the principle of treatment is basically the same, and surgical resection is considered the only effective treatment for mucoepidermoid carcinoma. Theoretically, the treatment principle of advanced patients who cannot be surgically resected is the same as that of NSCLC, with radiotherapy or chemotherapy, etc. Most scholars believe that postoperative adjuvant treatment is not necessary for low-grade PMEC, but postoperative radiotherapy or chemotherapy is recommended for patients with positive lymph nodes or positive tumor margins after surgery.^[10]^ However, postoperative radiotherapy or chemotherapy is recommended for patients with positive lymph nodes or positive tumor margins. Highly malignant mucoepidermoid carcinoma is prone to regional lymph node metastasis, and the prognosis of patients is generally poor. According to statistics, most PMECs show inert growth, but the prognosis of highly malignant PMECs is poor. Even if early surgery is performed, distant metastasis can still occur in a short period of time, and the 5-year survival rate is low.^[11]^ In recent years, molecular targeted therapy has become a research hotspot in the comprehensive treatment of lung cancer. MEC patients with mutations in the epidermal growth factor receptor (EGFR) gene have been found in approximately 40% of patients, and data suggest that the use of an EGFR-tyrosine kinase inhibitor (EGFR-TKI) in PMEC patients with EGFR mutations may improve survival.^[10]^ Macarenco reported that 92% of PMEC tissues were positive for EGFR expression, indicating a high mutation rate; however, some data reports in China show that EGFR gene mutations in PMEC are not rare, although in patients without EGFR gene mutations, clinical benefits may also be achieved with EGFR-TKI treatment, but a larger sample size is needed to support the data.^[12,13]^ In this case, EML4-ALK mutation was found in PMEC, and after the use of Loratinib ALK inhibitor, the symptoms were significantly reduced, and the tumor was significantly shrunken, which means that EML4-ALK may be a new research direction for the treatment of PEMC, and therefore, a more extensive clinical study should be conducted on targeted therapy, which is expected to be a breakthrough point for the treatment of PMEC.

## 4. Conclusion

In conclusion, this case suggests that the application of molecularly targeted drugs to patients with genetic mutations after genetic testing for EGFR and ALK can significantly improve the prognosis of patients with PMEC, which is expected to be a new hope in the treatment of pulmonary mucoepidermoid carcinoma. It is worth noting that the lack of large-scale clinical trials of molecularly targeted therapy for PMEC is not sufficient to predict the individualized treatment of targeted drugs, and the optimal target population, exact efficacy, dosage, and targeted drug resistance still need further research.

## Acknowledgments

We would like to thank the patient and her family.

## Author contributions

**Resources:** Yanyan Ren, Lijiao Yang.

**Supervision:** Xing Yin.

**Writing – original draft:** Lijiao Yang.

**Writing – review & editing:** Xing Yin.
